# Surgical Management of a Bullet Embolism to the Pulmonary Artery

**DOI:** 10.7759/cureus.8138

**Published:** 2020-05-15

**Authors:** Vitaley Kovalev, Oscar D Salaiz

**Affiliations:** 1 Acute Care Surgery, California Hospital Medical Center, Los Angeles, USA; 2 Basic Medical Sciences, College of Osteopathic Medicine of the Pacific, Western University of Health Sciences, Pomona, USA

**Keywords:** pulmonary embolism, bullet embolism, pulmonary artery, thoracotomy

## Abstract

Bullet embolism is a rare but potentially serious complication of a gunshot wound. This case report describes a 26-year-old male who presented with a gunshot to the lower back. Diagnostics revealed a migrating bullet that became lodged in the left pulmonary artery. After two unsuccessful attempts at endoscopic removal, the decision was made by the multidisciplinary team to retrieve the bullet surgically. The patient recovered well postoperatively. Four- and eight-month follow-up in the emergency department revealed no significant postoperative complications. Bullet embolism should be suspected when radiographs reveal a migrating projectile. Treatment options include conservative management, endoscopic bullet retrieval, and surgical removal. No guidelines for the management of a bullet embolism exist. Management should be based on the patient's clinical status and comorbidities, facility resources, and perceived risk of undergoing surgical retrieval of the bullet.

## Introduction

Pulmonary artery bullet embolism is a rare and life-threatening complication of penetrating projectile injury. Most injuries occur as a result of low-velocity and small-caliber gunshot wounds (GSWs) [1]. Bullet embolism can be either arterial or venous, but an arterial embolism is more common [2].

The first reported case of a pulmonary artery bullet embolism was reported by Moresten in 1903 [3]. Since then less than 200 cases have been described in the literature [4]. Bullet embolism is rare and occurred in 1.1% of 346 bullet injuries during the Afghanistan and Iraq wars [5]. Survival and complication rates have not been reported due to the rarity of this condition.

## Case presentation

**Figure 1:**
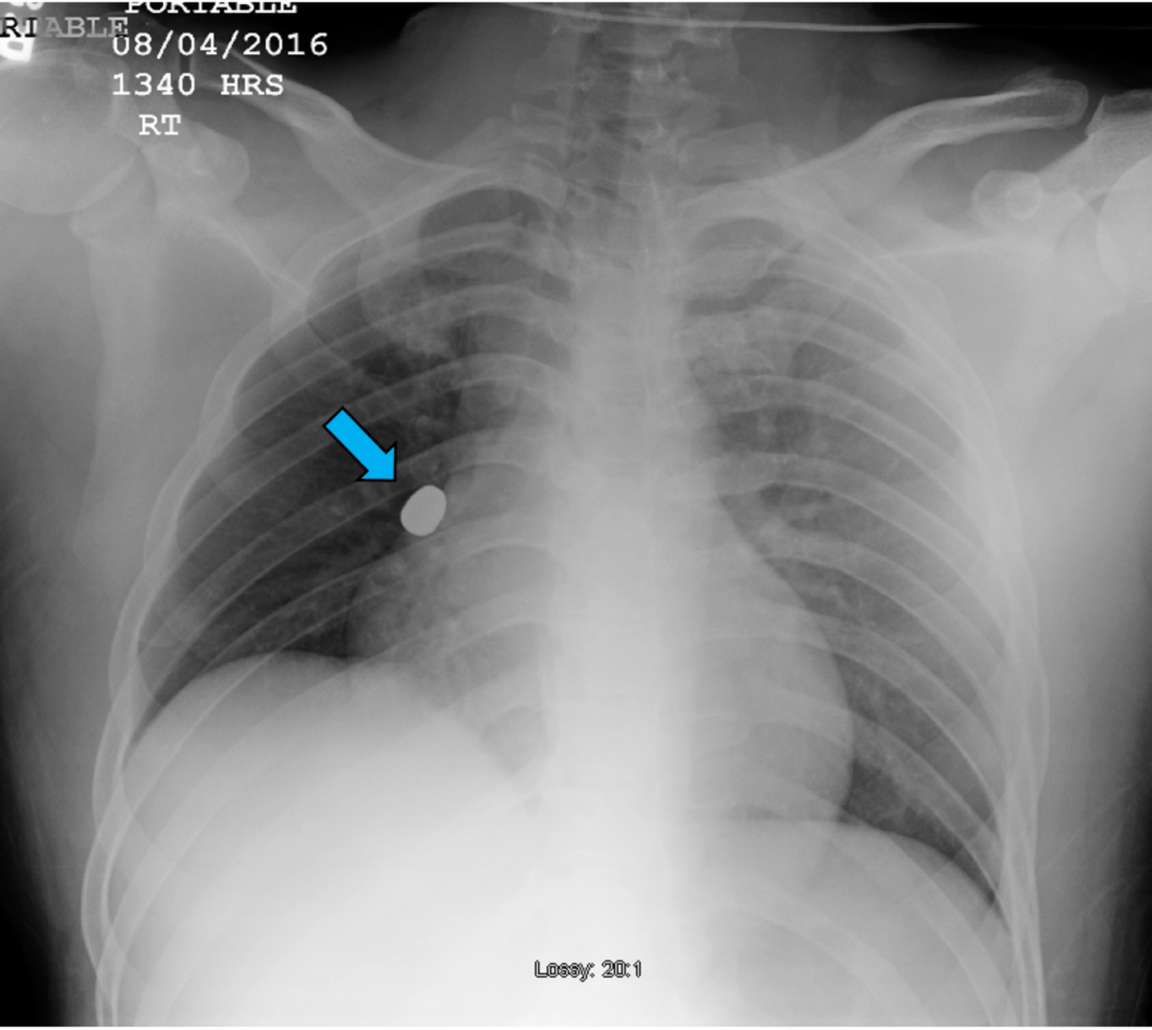
Supine chest radiograph, anterior-posterior view, with the bullet lodged likely in the right atrium (blue arrow).

A 26-year-old male was brought by ambulance to the emergency department (ED) after he sustained a single GSW to the lower back. Upon presentation, the patient was alert with a Glasgow Coma Scale of 15/15. His blood pressure was 107/38 mmHg, heart rate 110/min, respiratory rate 22/min, and oxygen saturation 98% on room air. On initial physical examination, he had a single penetrating injury just superior and to the left of the origin of the intergluteal cleft. Lungs were clear to auscultation bilaterally with no lesions noted on the chest; the abdomen was soft and non-tender. There was stool on the patient's clothing. However, he had good rectal sphincter tone with no gross blood on rectal examination. Neurological examination revealed 5/5 strength of both legs. The patient admitted to paresthesias to the left leg, but sensation of both legs was intact. There was mild restriction of active range of motion of the left hip due to pain. His dorsalis pedis pulses were 2/3. Blood pressure improved after one liter of crystalloid and one unit of packed red blood cells. His hemoglobin was measured at 8.8 g/dL. A chest and pelvic radiographs were obtained and revealed a bullet near the right pulmonary hilum (Figure 1). There was no pneumothorax or effusion noted on the chest radiograph and no foreign body or fracture on the pelvic radiograph.

**Figure 2:**
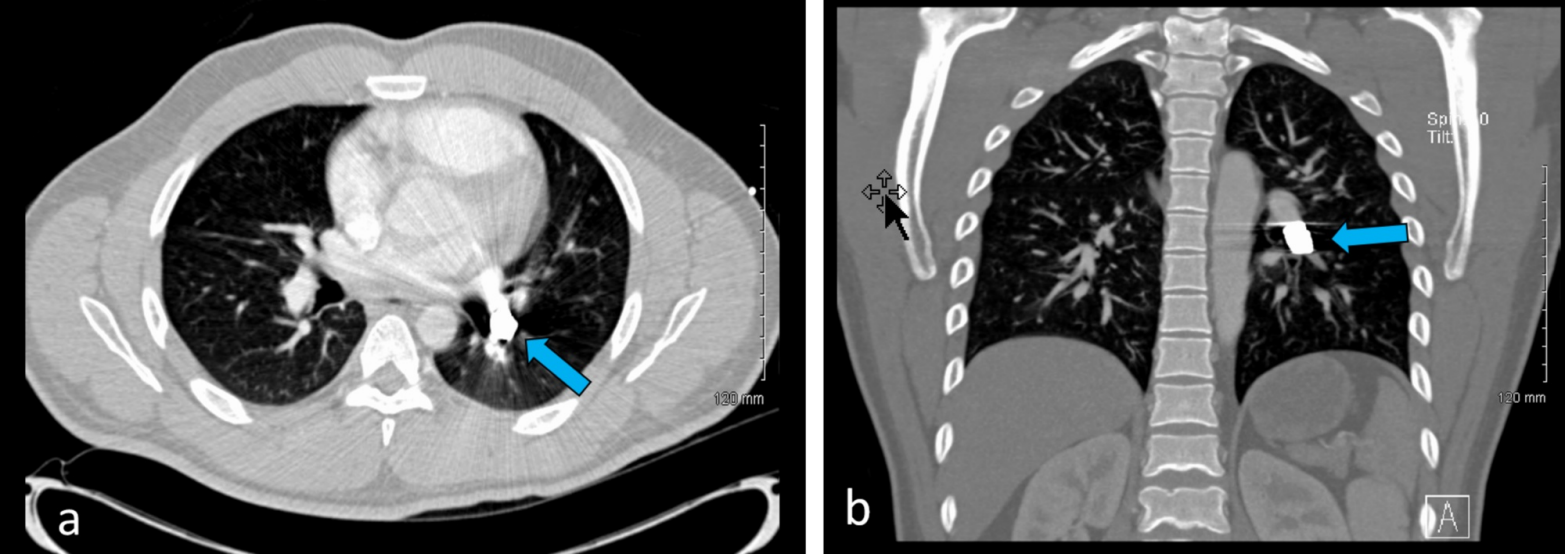
Computerized tomography of the chest with intravenous contrast. Bullet in the left pulmonary artery found on an axial view (a) and coronal view (b).

**Figure 3:**
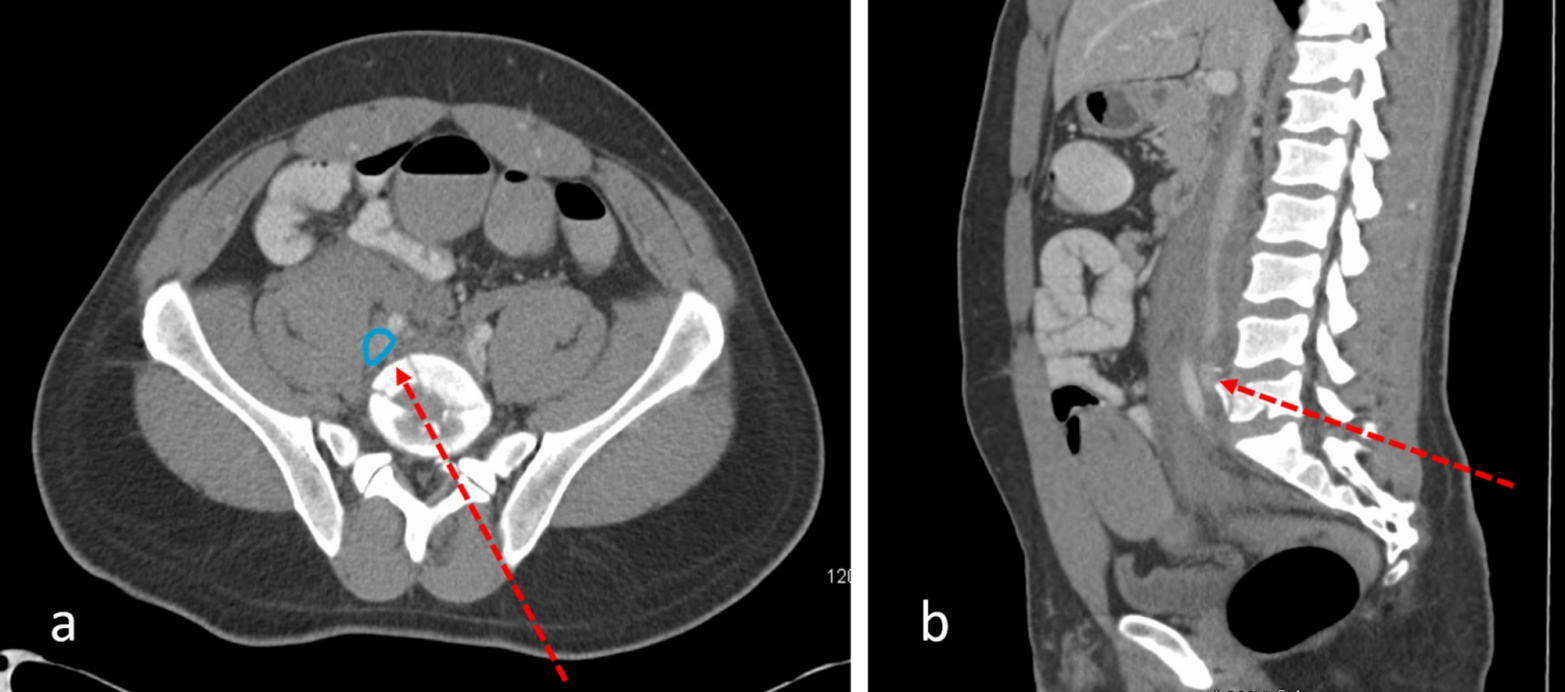
Computerized tomography of the abdomen and pelvis with intravenous contrast. Proposed trajectory of the bullet is entering the left lower back, through the L5 vertebral body (a, b, dashed red arrow) and then the right common iliac vein posteriorly (a, blue circle).

Twenty-three minutes later, he was then taken for computerized tomography (CT) with intravenous contrast of the chest, abdomen, and pelvis. His CT scans revealed a bullet fragment adjacent to the left hilum and left lower lobe pulmonary artery (Figure 2), shattered L5 and S1 vertebrae, tiny bullet and bone fragments within the spinal canal, possible injury to the inferior vena cava (IVC) or right common iliac vein, retroperitoneal hematoma (Figure 3), but no active extravasation of contrast. It was hypothesized that the projectile entered the posterior right common iliac vein through the lower back and embolized through the right side of the heart into the left pulmonary artery. Cardiothoracic surgery, interventional radiology (IR), and neurosurgery were immediately consulted. After a discussion of management options with the patient, it was decided to perform removal of the bullet. For the sacral and lumbar fractures neurosurgery recommended non-operative management. On hospital day 2, IR attempted an endovascular removal of the left lower pulmonary artery bullet with a large snare, but this was unsuccessful. A second attempt by IR was made the next day using various catheter techniques and different snares, but again this was unsuccessful. Consideration of open chest removal was given. Cardiothoracic surgery had a lengthy discussion with the patient and the family about the risks and benefits of the procedure. Because the patient was young and descriptions of long-term complications of conservative management of a bullet embolus are sparse, the decision was made to perform an open chest removal of the bullet. On hospital day 4, the patient was taken to the operating room by cardiothoracic surgery for bullet retrieval. The bullet removal was accomplished via a posterolateral thoracotomy incision. The left main pulmonary artery was identified, a longitudinal opening was made in the distal portion, and the bullet was identified and extracted from the distal left lower pulmonary artery. The patient was moved back to the intensive care unit in stable condition and started on a prophylactic dose of unfractionated heparin with bridging to coumadin. He was extubated on postoperative day 2. Once the patient's international normalized ratio was in the therapeutic range, the patient was discharged home with a plan to continue coumadin for three months. 

Notably, the patient returned to the ED four months later with left chest wall pain at the location of the thoracotomy incision. A CT angiogram (CTA) was obtained and showed early truncation of the left lower basilar pulmonary artery segment with non-filling of the distal segmental arteries but no distal pulmonary infarct. No evidence of pulmonary embolism (PE) was found. There was a soft tissue filling defect along the posterior aspect of the left main pulmonary vein with mild enlargement representing non-mixing of venous blood or possible venous thrombus. At this point, he had already completed his three-month coumadin regimen. Cardiology was consulted and, because of his complicated medical history, recommended that the patient is started on rivaroxaban 15 mg twice per day for 21 days and then 20 mg once per day for three months. The patient once again returned to the ED four months later with the same left chest wall pain, which was deemed to be incisional. A CTA was performed again and showed chronic fibrotic narrowing of the left lower lobe pulmonary artery with no evidence of PE. He was discharged with primary care follow-up.

## Discussion

Bullet embolism occurs when a projectile penetrates vasculature and migrates into the pulmonary arterial tree [6,7]. Typical entry sites of the GSW are proximal lower extremity, buttock, back, abdomen, and neck [6]. Entrance to the vasculature may be through venous or arterial access [8]. The mechanisms causing tissue damage are through laceration, crush, shock wave, and cavitation injuries [9]. Once the bullet enters the vasculature migration generally occurs in the direction of blood flow until the fragment is obstructed or becomes lodged in the pulmonary arterial tree as in the case [10]. At this juncture, pulmonary and systemic consequences may be in the form of mechanical obstruction, causing ischemia, or due to the nature of the material and contamination, which may be a nidus for infection [6,8,11]. 

Recognizing a bullet embolism in a patient suffering from a GSW is challenging. In the trauma setting, it is imperative to account for the number of penetrating wounds and corresponding exit wounds on physical exam. When there are a greater number of entrance wounds compared to exit wounds, there must be a high level of clinical suspicion to locate the retained bullet or fragments [10]. X-ray is often the initial means for the detection of a retained bullet from a GSW. We suggest that as soon as a migrating projectile is noted on X-ray and a bullet embolism is suspected, the patient be placed in an upright position, if circumstances allow, to prevent migration of the projectile into the pulmonary vasculature. CT can confirm the presence and location of the bullet [12]. MRI is contraindicated in such patients due to potential risk of causing further damage from strong magnetic forces [13]. The presentation of bullet embolism varies tremendously from asymptomatic to sudden death [6]. In one review, up to 70% of retained bullet materials in venous vasculature were asymptomatic [14]. Conversely, arterial involvement presents with symptomatic ischemia in a majority of cases [10].

Management of patients with bullet embolism depends on the severity of their presentation, hemodynamic stability, and perceived risk of complications. Emergent or urgent retrieval of the bullet may be warranted initially through an endovascular approach. If this is contraindicated or unsuccessful, removal through surgical thoracotomy is the next option [6]. In some cases, such as an asymptomatic patient, simple observation might be a better course of action. In one analysis of 32 cases of bullet embolism from 1966 to 1992, five patients who were managed conservatively and were evaluated at the nine-month follow-up demonstrated no complications due to the retained bullet [15]. Although the mentioned study is decades old and albeit a small sample size, publications such as this are extremely sparse and with no current guidelines available to aid in decision making. Management of bullet embolism remains controversial.

Prognosis of bullet embolism is rarely fatal, and often patients continue to be asymptomatic. However, serious complications, including infection, thrombosis, ischemia, hemorrhage, inflammatory reactions, and even death, have occurred [6,11]. These complications depend on the location of GSW, trajectory, extent of damage, comorbidities, and need of resuscitation on presentation or during any procedures [6].

The presented case is unique for several reasons. Aside from a retroperitoneal hematoma near the psoas, there was no internal hemorrhage or damage to vital organs. Despite a large bullet fragment lodged in the left pulmonary artery, there was no respiratory compromise. Since the patient was young with limited long-term outcomes documented in the literature along with the patient’s wishes for the removal of the bullet, a decision was made to treat surgically. Another important feature of the case is the information gained from four- and eight-month follow-up visits to the ED which demonstrated no evidence of PE on CTA.

## Conclusions

Bullet embolism is a rare but potentially serious complication of GSW. The astute practitioner should have a high index of suspicion when the number of entry wounds is greater than the number of exit wounds. A chest X-ray can screen for a retained bullet, while CT can confirm its location. Treatment options include conservative management, endoscopic bullet retrieval, and surgical removal. Management should be based on clinical acumen, facility resources, hemodynamic state of the patient, patient’s comorbidities, and perceived risk of undergoing surgical retrieval of the bullet.
